# Minimally Invasive Management of Vesicovaginal Fistulae: Laparoscopic Experience From a Teaching Hospital in Northeastern Mexico

**DOI:** 10.7759/cureus.90002

**Published:** 2025-08-13

**Authors:** Jose Luis Maldonado-Calderón, Jose Antonio Zapata-Gonzalez, Luis Enrique Torres-Zapata, Jesus Garcia-Saucedo, Adrian Gutierrez-Gonzalez, Samantha B Medrano-Juarez

**Affiliations:** 1 Urology, Hospital Universitario Dr. José Eleuterio González, Universidad Autónoma de Nuevo León, Monterrey, MEX

**Keywords:** hysterectomy complications, laparoscopic surgery, minimally invasive repair, urogenital fistula, vesicovaginal fistula

## Abstract

Introduction

Vesicovaginal fistula (VVF) is an abnormal communication between the bladder and vagina that leads to continuous involuntary urine leakage and profoundly affects quality of life. In developed countries, most cases are iatrogenic, often following hysterectomy. Laparoscopic repair has emerged as a minimally invasive option with promising outcomes. This study aims to describe the experience of a tertiary referral center with laparoscopic repair of VVF.

Methods

A retrospective descriptive study was conducted at a university hospital in Monterrey, Mexico, between March 2023 and March 2025. Seven patients who underwent laparoscopic VVF repair were included. Preoperative evaluation involved clinical, imaging, and endoscopic assessment. All repairs were performed using the O’Conor technique with omental interposition. Perioperative variables and outcomes were analyzed.

Results

All VVFs were secondary to gynecological surgery (six abdominal hysterectomies and one vaginal hysterectomy). The mean patient age was 48 ± 6.2 years, with a BMI of 29.3 ± 3.8 kg/m². The mean time from hysterectomy to repair was 14.7 ± 8.6 months. Six fistulas were classified as simple and one as complex. Mean fistula size was 20 (15.0 - 20.0) mm, and 71.42% were supratrigonal. Mean operative time was 235.71 ± 155.52 minutes, with no conversions to open surgery. The average hospital stay was 2.7 ± 1.38 days, and Foley catheter removal occurred at 20.86 ± 3.53 days. No intraoperative or postoperative complications were observed, and no recurrences were noted during a mean follow-up of 2.57 ± 0.79 months.

Conclusion

Laparoscopic repair of VVF is a safe and effective surgical option, with favorable outcomes and minimal morbidity, particularly when standardized surgical principles are applied and omentum is used as an interpositional tissue. Further prospective studies are needed to define its role in broader clinical practice

## Introduction

A fistula is defined as an abnormal communication between two epithelialized surfaces. In the urinary tract, fistulas involve a pathological connection between the urinary system and adjacent anatomical structures [1]. Among urogenital fistulas, the most common type is the vesicovaginal fistula (VVF), which causes continuous and involuntary leakage of urine into the vaginal canal - an issue that significantly impairs patients’ quality of life [2].

The etiology of VVF varies depending on geographic location. In developing countries, the primary cause is prolonged obstructed labor, which leads to pressure necrosis in the pelvic floor, anterior vaginal wall, and bladder trigone [1]. In contrast, in developed countries, gynecological surgery is the leading cause of VVF, with hysterectomy being the most common surgical procedure associated with its development, with a reported prevalence of 0.1-0.2% [1,3].

Diagnosis is often clinical; however, imaging studies such as cystography, delayed-phase contrast-enhanced computed tomography (CECT), and endoscopic procedures including cystoscopy and vaginoscopy are essential for accurate documentation of the fistula [4].

Treatment options range from conservative management and endoscopic techniques to transvaginal and transabdominal surgical approaches. Among transabdominal approaches, minimally invasive techniques have increasingly gained prominence in the surgical repair of VVF [5]. The aim of this study is to describe our institution’s experience with the laparoscopic repair of VVFs.

## Materials and methods

This retrospective descriptive study was conducted at Hospital Universitario Dr. José Eleuterio González, a teaching hospital in Monterrey, Mexico, between March 2023 and March 2025. Institutional ethical approval was obtained prior to data collection. The medical records of all female patients who underwent laparoscopic VVF repair during this period were retrospectively reviewed using the following inclusion criteria: female patients ≥ 18 years of age, diagnosis of VVF confirmed both clinically (continuous urinary leakage through the vagina) and radiologically (CT cystography and/or cystoscopy), having undergone laparoscopic fistula repair, and having a complete medical history available. Exclusion criteria were as follows: history of cervical or pelvic malignancy, prior pelvic radiotherapy, presence of concomitant urogenital fistulas (ureterovaginal, rectovaginal), or incomplete clinical or imaging data.

Data were collected from electronic medical records. Preoperative evaluation included clinical history, physical examination, complete blood count, serum chemistry, and delayed-phase intravenous CECT to assess upper urinary tract integrity. Cystoscopy and vaginoscopy were also performed intraoperatively, prior to the start of the laparoscopic procedure.

Clinical and surgical variables were analyzed, including previous surgical history; fistula size, multiplicity, and location; need for conversion to open surgery; operative time; estimated blood loss; postoperative complications; length of hospital stay; time to Foley catheter removal; and postoperative urinary incontinence. A voiding cystogram was performed prior to the definitive removal of the Foley catheter. Postoperative follow-up was conducted for at least two months. Patients with positive urine cultures received preoperative antibiotic therapy.

Statistical analysis was performed using IBM SPSS version 20 (IBM Corp., Armonk, NY, USA). Patient data were anonymized to preserve confidentiality. Data cleaning and coding were carried out prior to analysis. Continuous variables were reported as mean ± standard deviation (SD) or median with interquartile range (IQR), depending on the distribution. Categorical variables were expressed as frequencies and percentages. The normality of the data was assessed using the Shapiro-Wilk test.

Surgical technique

Step One: Patient Positioning

Under general anesthesia, the patient was placed in a modified Lloyd-Davies position with the lower limbs supported by adjustable stirrups. Elastic compression bandages were applied to the lower extremities as a prophylactic measure against thromboembolism. The perineal area was prepared and draped in a sterile manner, ensuring access for transvaginal intervention if required.

Step Two: Cystoscopy and Ureteral Catheterization

A diagnostic cystoscopy was performed using a 22 Fr sheath and a 30° lens. The fistulous tract was identified and cannulated using a Bentson-type hydrophilic guidewire. Both ureteral orifices were catheterized with 6 Fr open-end ureteral catheters, which were externalized through a 20 Fr Foley catheter placed transurethrally. Vaginal gauze packs were inserted to occlude the introitus and prevent carbon dioxide leakage during the laparoscopic procedure.

Step Three: Trocar Placement

A 10 mm supraumbilical trocar was inserted using the open Hasson technique for laparoscopic optics. Under direct vision, two or three 5 mm trocars were placed in a diamond configuration to optimize triangulation and access to the pelvic cul-de-sac.

Step Four: Adhesiolysis and Pelvic Dissection

**Figure 1:**
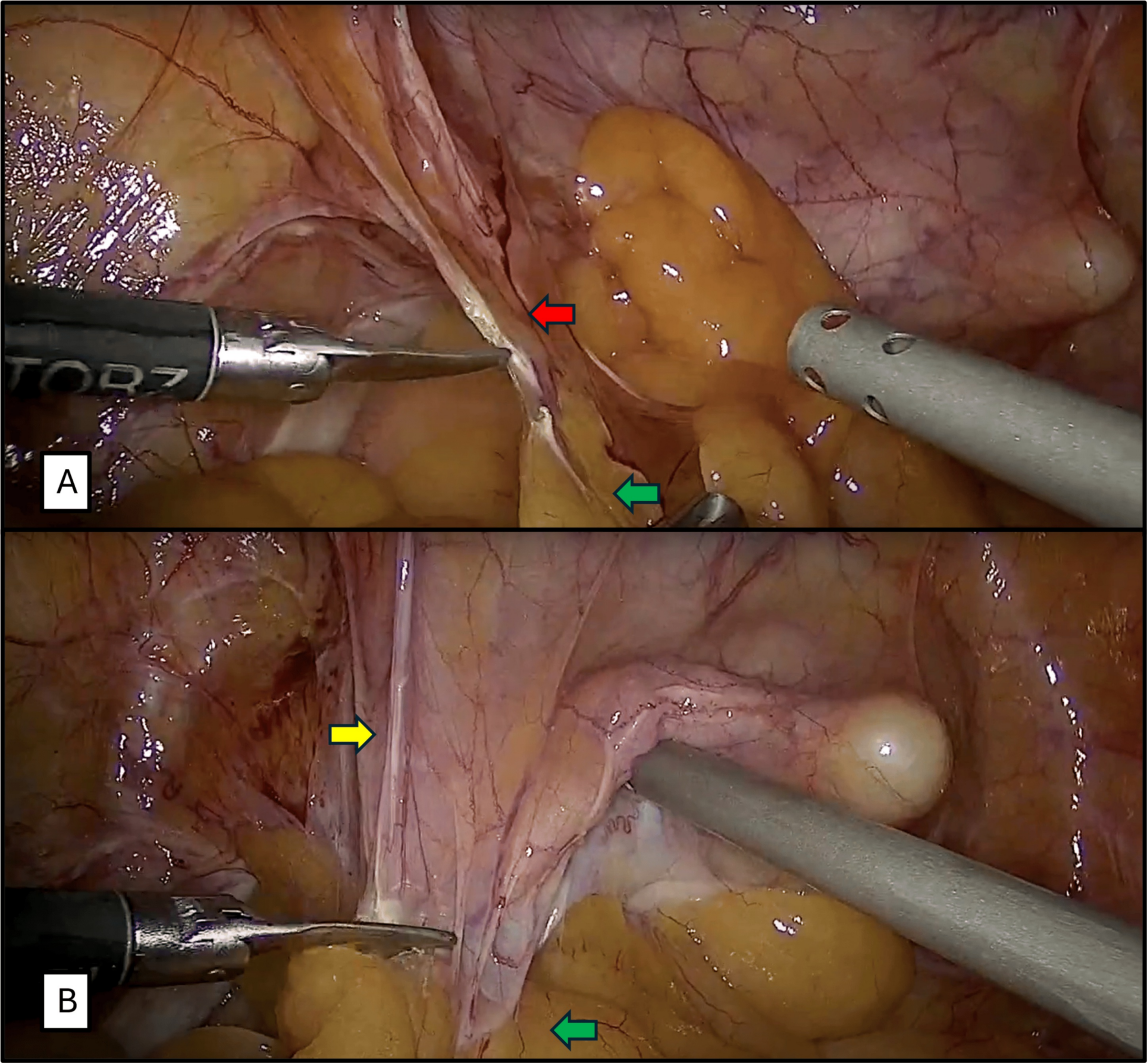
Adhesiolysis. A. Cutting of loose adhesions between the abdominal wall and the sigmoid colon using monopolar energy and cold cutting (Red arrow: loose adhesion. Green arrow: sigmoid colon). B. Release of adhesions between the posterior surface of the bladder and the sigmoid colon using monopolar energy and cold cutting (Yellow arrow: posterior face of the bladder. Green arrow: sigmoid colon).

Upon entering the peritoneal cavity, adhesions between bowel loops and between the bowel and bladder-particularly in the anterior cul-de-sac-were carefully released. This step allowed adequate exposure of the vaginal apex and the fistulous tract (Figure 1). 

Step Five: Resection of the Fistulous Tract

**Figure 2:**
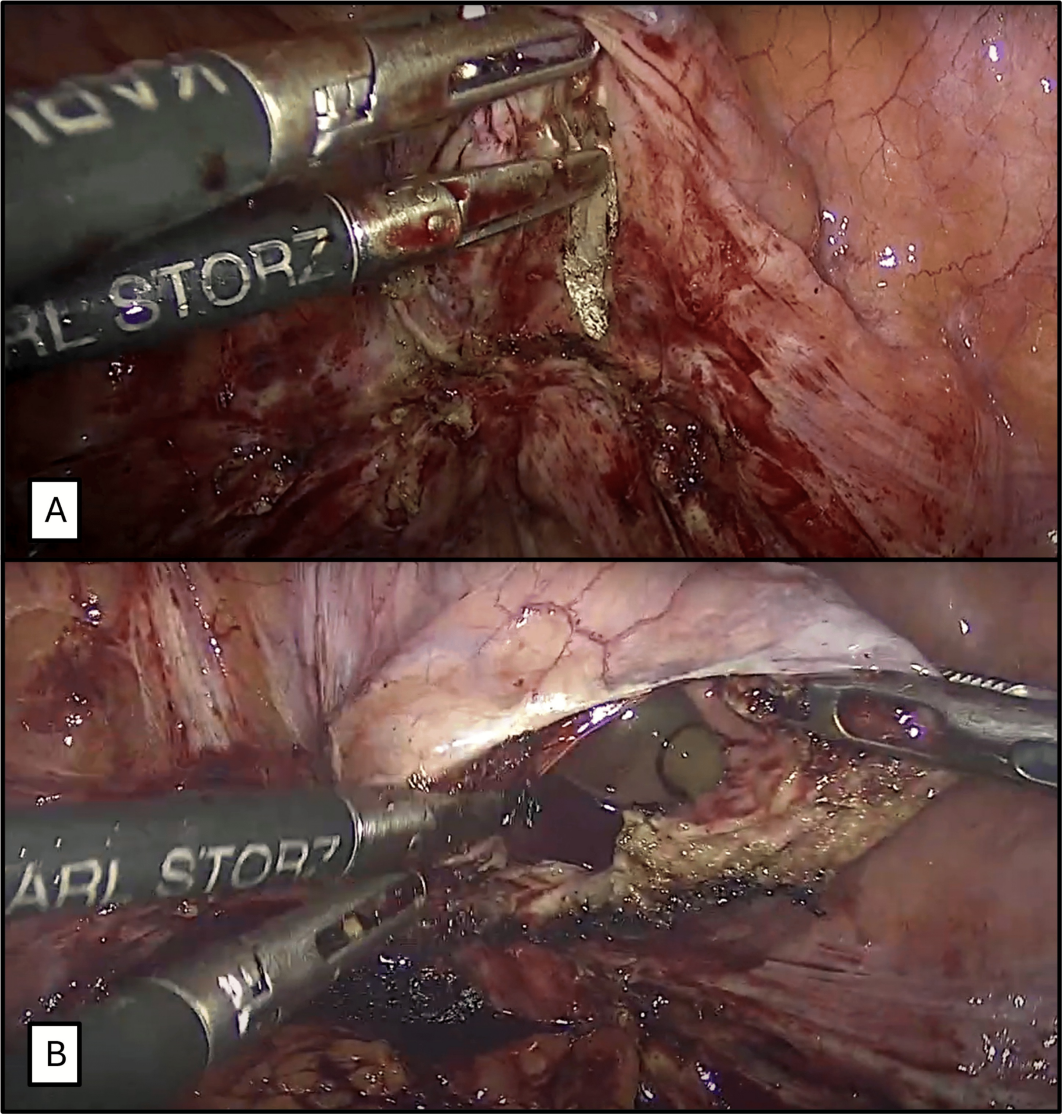
Cystotomy. A. A vertical cystotomy is performed on the posterior bladder wall using monopolar energy. B. Transurethral Foley catheter is seen inside the bladder through the cystotomy.

**Figure 3:**
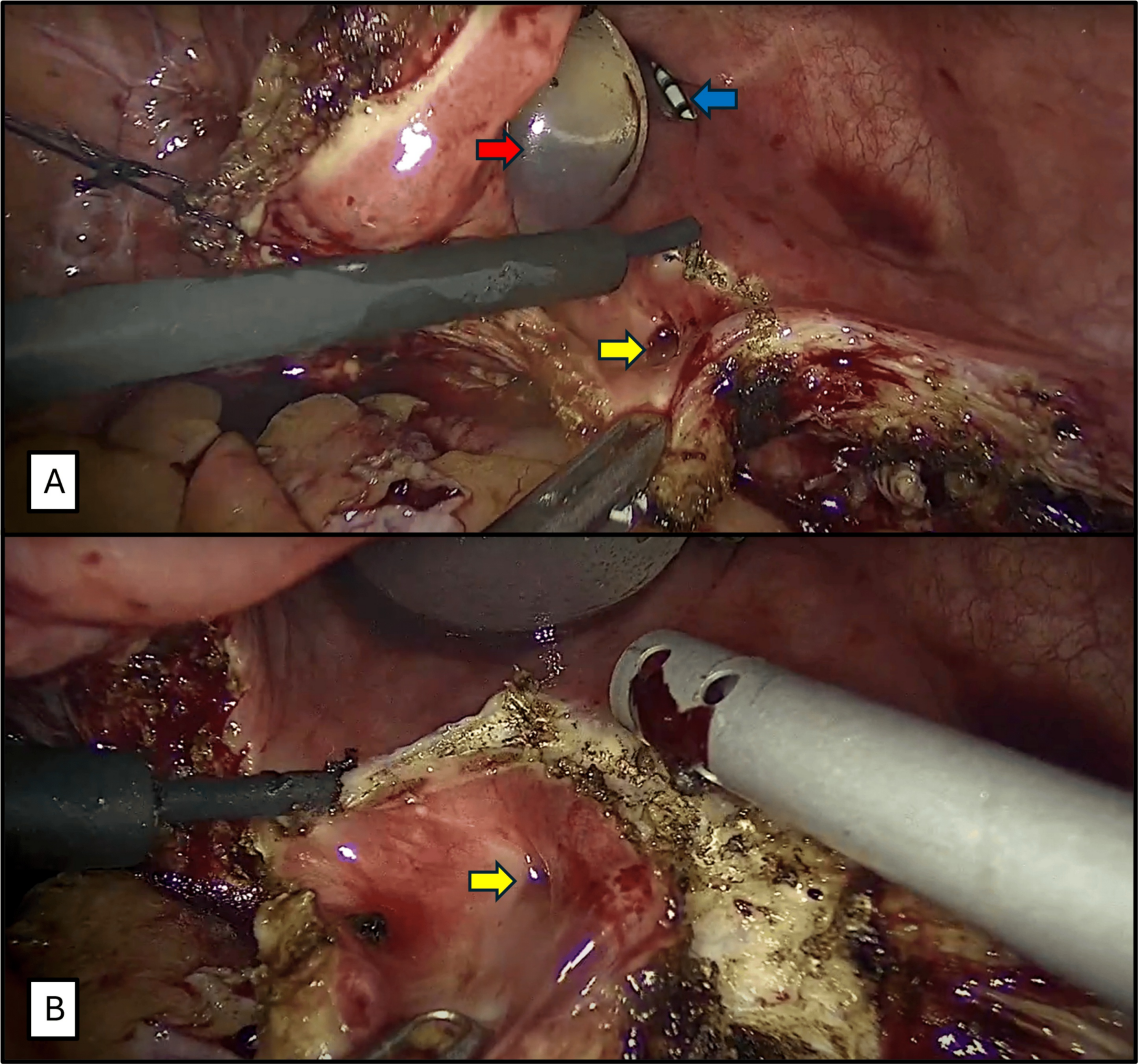
Resection of the fistulous tract. A. Marking the fistulous tract to be resected using monopolar energy with the hook (Yellow arrow: fistulous tract. Red arrow: balloon of the Foley catheter. Blue arrow: open end catheter at the right ureteral orifice). B. Resecting the fistulous tract using monopolar energy with the hook (Yellow arrow: fistulous tract).

A vertical cystotomy was performed on the posterior bladder wall to fully expose the fistulous opening. The fistulous tract was carefully dissected and resected using monopolar electrocautery, removing all scarred and necrotic tissue to minimize the risk of recurrence (Figures 2, 3).

Step Six: Vaginal and Bladder Closure

**Figure 4:**
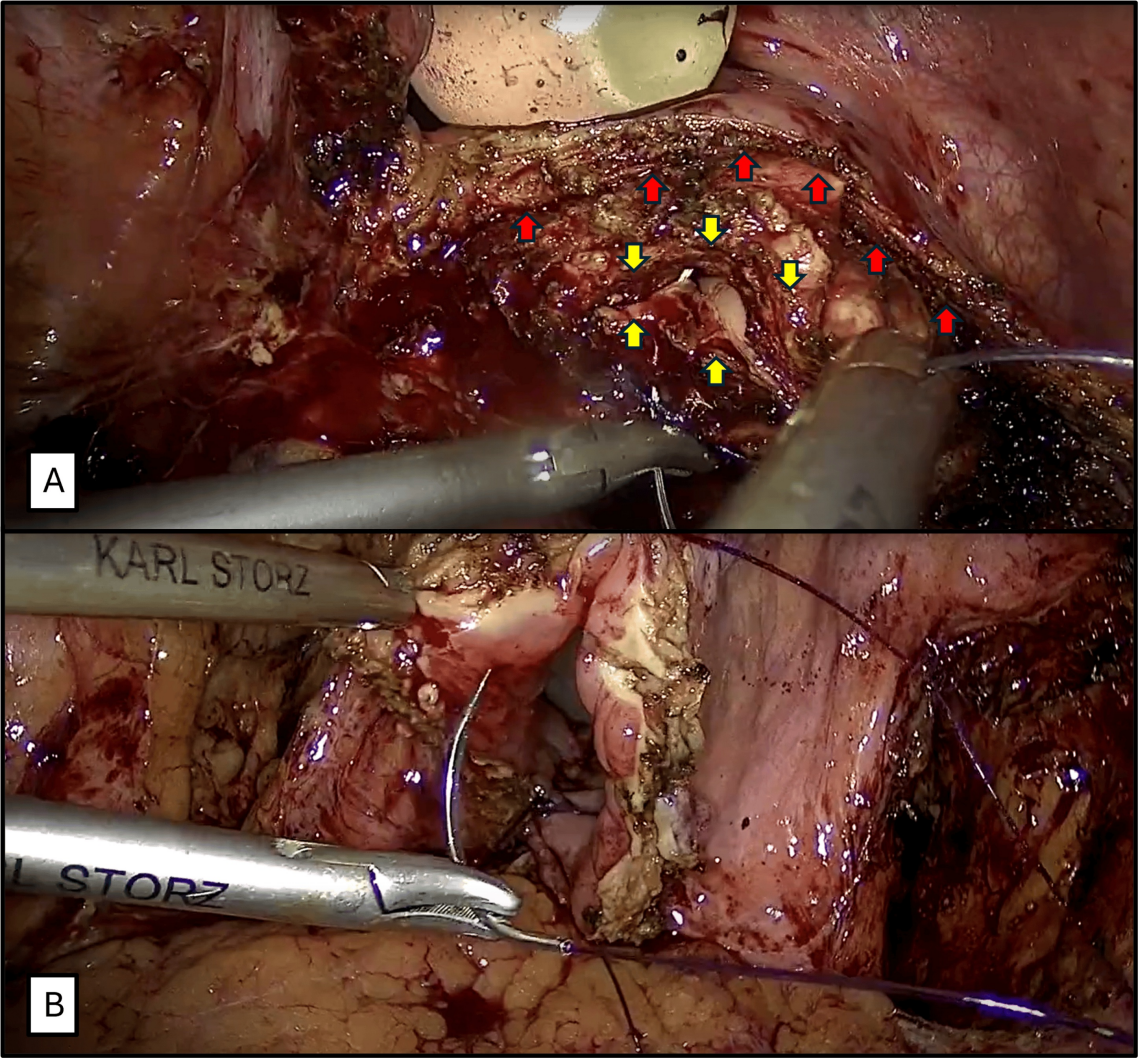
Vaginal and bladder closure. A. Closure of the vaginal defect with continuous suture (Red arrows: bladder wall. Yellow arrows: vaginal defect). B. Closure of bladder wall.

Vaginal repair was performed with a continuous suture using 2-0 Monocryl. The bladder was closed independently in a single continuous layer using absorbable suture (3-0 Vicryl). Both closures were performed in separate planes, ensuring a watertight and tension-free repair (Figure 4).

Step Seven: Omental Interposition

**Figure 5:**
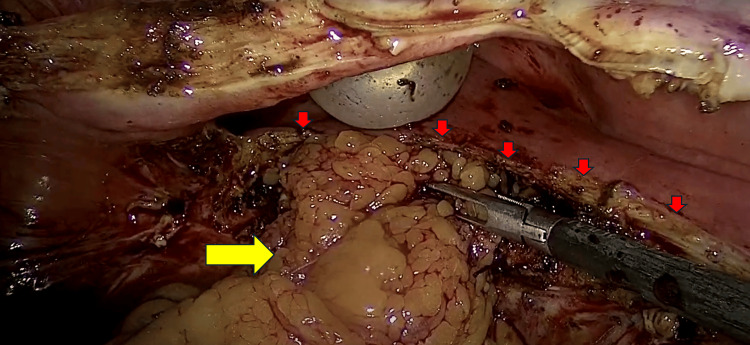
Omental interposition. An omental flap is placed and sutured in the plane between the vaginal repair and cystotomy closure (Yellow arrow: omental flap. Red arrows: Bladder wall).

A flap of the greater omentum was mobilized and interposed between the bladder and vagina, it was secured to adjacent pelvic structures using interrupted sutures of 3-0 Vicryl. This maneuver provides a well-vascularized barrier, enhances healing, and reduces the risk of recurrence (Figure 5).

Step Eight: Drain Placement and Port Closure

A 19 Fr Blake drain (or Penrose drain, depending on availability) was placed and directed toward the repair site. Hemostasis was confirmed under direct vision at all working sites. Port sites were closed using absorbable sutures (0 or 1 Vicryl) for the fascia, and subdermal sutures or skin adhesive were used for the skin closure, according to surgeon preference.

## Results

Between March 2023 and March 2025, seven laparoscopic fistulectomies were performed at our center. All fistulas were secondary to gynecological surgery: six following abdominal hysterectomy and one following vaginal hysterectomy. The mean age of the patients was 48 ± 6.2 years, with an average BMI of 29.3 ± 3.8 kg/m². The mean time from hysterectomy to fistula repair was 14.7 ± 8.6 months. Four patients had received prior conservative management. Six patients presented with simple fistulas, while one patient had undergone two previous repair attempts, classifying her case as complex. The average fistula size was 20 (15-20 IQR) mm. In all cases, the O’Conor technique was used for surgical repair. The characteristics of the fistulas are summarized in Table 1.

**Table 1:** Clinical characteristics of the patients and fistula details. Results are presented as number and percentage, mean ± standard deviation (SD), or median with interquartile range (IQR), as appropriate according to data distribution.

Clinical characteristics	N=7 (%)
Age (years)	
	48.0 ± 6.2
BMI (kg/m²)	
	29.3 ± 3.8
Cause of the fistula	
Hysterectomy	7 (100%)
Other	0 (0%)
Conservative management with bladder drainage	
Yes	4 (57.14%)
No	3 (42.86%)
Time from hysterectomy to fistulectomy (months)	
	14.7 ± 8.6
Fistula size (mm)	
	20.0 (15.0 - 20.0)
Fistula location	
Trigonal	2 (28.57%)
Supratrigonal	5 (71.42%)

In all cases, tissue transposition with omentum was performed. The mean operative time was 235.71 ± 155.52 minutes, and the average intraoperative blood loss was 97.86 ± 94.86 cc. No blood transfusions were required, and none of the procedures required conversion to open surgery. The mean postoperative hospital stay was 2.7 ± 1.38 days, and the average time from the procedure to transurethral Foley catheter removal was 20.86 ± 3.53 days. No postoperative complications were observed, and no patient exhibited urinary leakage during the voiding cystogram. The mean follow-up period was 2.57 ± 0.79 months. At the end of follow-up, no patient presented with urinary leakage (Table 2).

**Table 2:** Intraoperative and postoperative outcomes. Results are presented as number and percentage, or mean ± standard deviation (SD), depending on the type and distribution of the data.

Patient	Operative time (min)	Intraoperative bleeding (mL)	Surgical success rate	Postoperative hospital stays (days)	Time to removal of urinary catheter (days)	Duration of postoperative follow-up (months)
1	480	300	Yes	2	21	4
2	205	50	Yes	2	21	2
3	285	70	Yes	5	26	3
4	390	100	Yes	4	21	2
5	90	60	Yes	3	21	2
6	110	5	Yes	2	22	2
7	90	100	Yes	1	14	3
Mean±SD	235.71± 155.52	97.86 ± 94.86	7 (100%)	2.71 ±1.38	20.86 ± 3.53	2.57 ± 0.79
N=(%)						

## Discussion

Vesicovaginal fistula is a medical condition that significantly impacts patients' quality of life, with profound social, emotional, economic, and psychological consequences [5,6]. In industrialized countries, gynecological surgery - particularly hysterectomy - is the leading cause of these fistulas, which aligns with our findings, as 100% of the patients developed the injury following such a procedure [2,5]. In contrast, in developing regions, the most frequent cause remains prolonged labor, typically associated with pressure necrosis [5].

Clinically, patients present with continuous urinary leakage through the vagina, usually appearing one to two weeks after gynecological or pelvic surgery. The severity of urinary incontinence correlates with the size of the fistulous tract [2,7]. Persistent urine loss may lead to complications such as dermatitis, recurrent vaginal infections, cystitis, and even chronic pelvic pain [8].

**Figure 6:**
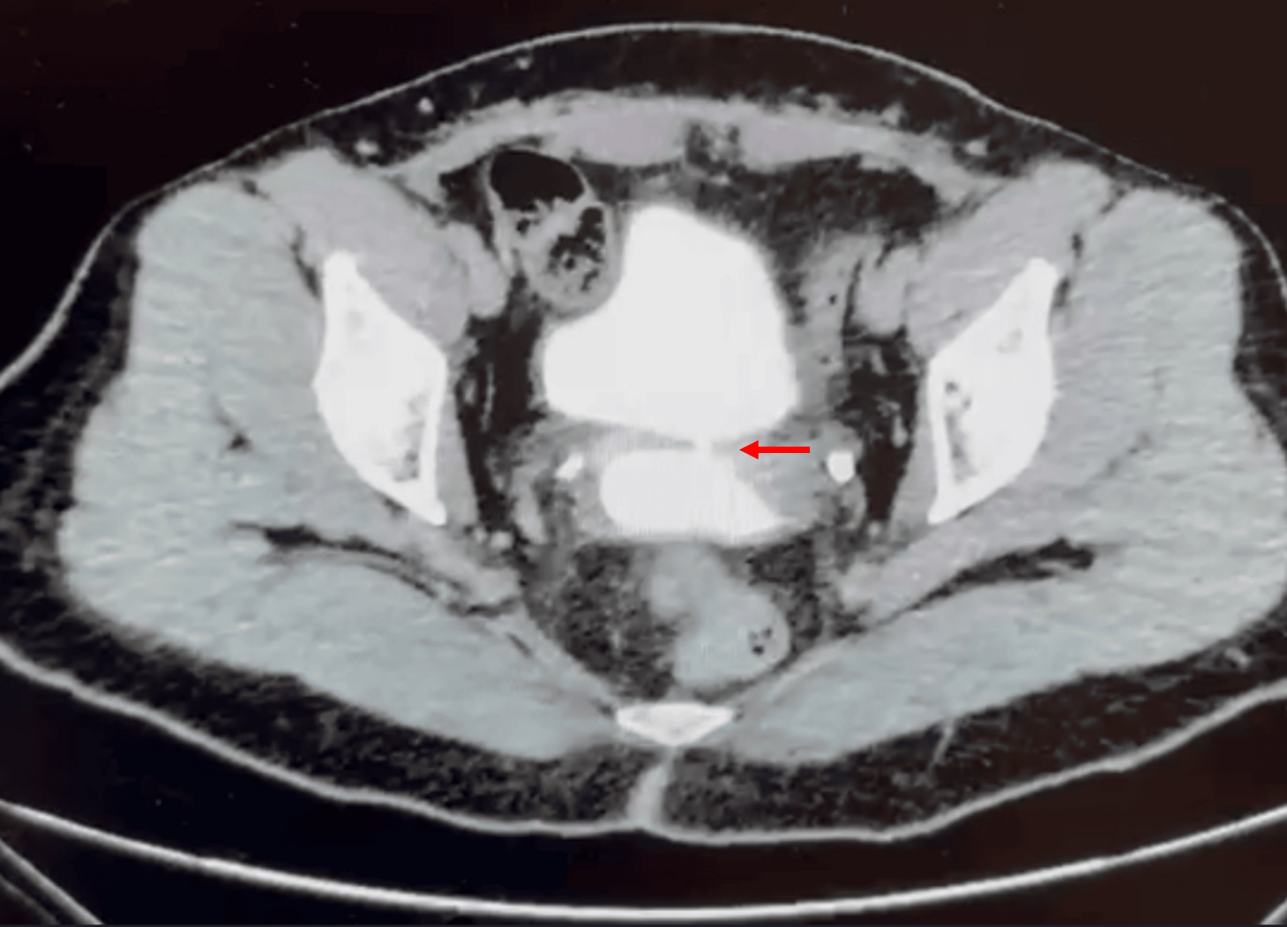
Axial delayed-phase contrast-enhanced computed tomography (CECT) showing a vesicovaginal fistula. Axial CT scan with intravesical contrast showing a 2 mm vesicovaginal fistulous tract (red arrow), with intravesical contrast passing through the fistula to the vagina of one of the patients included in the study.

A comprehensive diagnostic approach is essential and should include physical examination, cystoscopy, and vaginoscopy, along with complementary tests such as intravesical instillation of methylene blue or the placement of intravaginal gauze. Cystography may be useful to delineate the fistulous tract. It is also critical to assess the upper urinary tract, as approximately 12% of iatrogenic cases may present with concomitant ureteral injury [7]. In our center, all patients underwent delayed phase CECT, as well as CT-cystography to define the location, size, and path of the fistula. Additionally, cystoscopy and vaginoscopy were performed preoperatively (Figure 6).

Although at least seven classifications exist for vesicovaginal fistulas, the most commonly used system is based on size and the characteristics of the surrounding tissues [9]. Fistulas are considered complex if they exceed 3 cm, are recurrent, have undergone prior surgical attempts, or are associated with ureteral or rectovaginal injuries, prior radiation, or proximity to the continence mechanism or urethral meatus [10]. Among the seven patients included, the mean fistula size was 20 (15.0 - 20.0 IQR) mm, and most of the fistulas (71.42%) were supratrigonal. Six cases were classified as simple fistulas, while one was considered complex due to a history of two failed surgical repair attempts.

Conservative management with bladder drainage may be initially considered, however, there is no conclusive evidence linking the duration of catheterization with the likelihood of spontaneous closure [11]. If the fistula persists after 30 days of drainage, surgical repair is recommended, as prolonged catheter use increases the risk of infection [7]. In our study, four patients received initial conservative management with a Foley catheter prior to definitive surgical intervention.

Surgery remains the gold standard for VVFs that do not resolve with conservative treatment [10]. One of the most debated aspects in the literature is the optimal timing for surgical repair. If the fistula is identified within the first 72 hours, immediate repair may be attempted. However, in most cases, it is recommended to delay surgery for at least three months to allow resolution of inflammation and optimize outcomes [5,12]. More recent proposals suggest an individualized approach, taking into account factors such as fistula type, etiology, and the patient’s clinical condition [1]. In our cohort, the average time from hysterectomy to surgical repair was 14.7 ± 8.6 months.

Surgical approaches may be transvaginal or transabdominal, with comparable success rates. The choice depends on fistula characteristics and the surgeon’s expertise [13]. Laparoscopic repair was first described by Nezhat et al. in 1994 [14] and has since gained popularity. In 2005, Sotelo et al. reported 13 laparoscopic repairs, with only one recurrence [15]. Subsequently, Porpiglia et al. in 2009 described successful closure in four patients via a transvesical approach [16]. In Mexico, Zapata-González et al. reported a series of 19 cases with an 89.4% success rate, while Ríos-Melgarejo et al. reported a 93.3% success rate in 15 patients treated laparoscopically [10,17].

Successful surgical repair requires adherence to key principles: adequate exposure of the fistula, excision of the tract, watertight tension-free closure, layered cystorrhaphy, interposition of healthy, well-vascularized tissue between the vagina and bladder, and effective postoperative bladder drainage [10]. In all cases, we used omentum as the interposed tissue, which we consider a key factor contributing to our high success rate, consistent with the findings of Porpiglia and Sotelo, however, other vascularized tissues can be used as peritoneum or epiploic appendages [15,16]. Among the most relevant prognostic factors for successful repair are fistula size (<2 cm) and availability of interpositional tissue, both of which were present in our patients [17,18].

With regard to postoperative care, bladder drainage is recommended for at least 14 days and may be extended up to three weeks to ensure proper healing. Prior to catheter removal, a voiding cystogram should be performed to confirm complete fistula closure [5,8,9]. In our series, the average duration of catheterization was 20.86 ± 3.53 days, and in all cases, closure was confirmed via cystography before removal.

Limitations

This study has several limitations. First, it is a retrospective single-center experience with a small sample size, which limits the generalizability of the findings. Second, the follow-up period was relatively short, and therefore long-term recurrence rates could not be assessed. Finally, patient-reported outcomes such as quality of life were not evaluated, which could have provided additional insights into the clinical impact of the procedure.

## Conclusions

Laparoscopic repair is an effective, safe, and minimally invasive option for the management of vesicovaginal fistulas secondary to gynecological surgery. Our findings support its use, demonstrating high success rates and low complication risks, particularly when standardized surgical principles are applied and omentum is used as an interpositional tissue. Although current evidence is promising, prospective and comparative studies are still needed to better define the role of laparoscopy and to establish evidence-based recommendations for its use across various clinical settings.

## References

[1] Nigro N, Shahinyan G, Lin S, Bhalla RG, Flynn BJ (2025). A comprehensive review of urinary tract fistulas: the evolution of etiologies, surgical techniques, and contemporary outcomes. Ther Adv Urol.

[2] AlQattan T, AlMehzaa R, Qureshi A, AlSary S (2025). Laparoscopic repair of a vesicovaginal fistula after total abdominal hysterectomy. Cureus.

[3] Simforoosh N, Soltani MH, Lashay A, Ojand A, Nikkar MM, Ahanian A, Sharifi SH (2012). Laparoscopic vesicovaginal fistula repair: report of five cases, literature review, and pooling analysis. J Laparoendosc Adv Surg Tech A.

[4] Fouladi DF, Shayesteh S, Fishman EK, Chu LC (2020). Urinary bladder fistulae and the role of CT cystography: a pictorial review. Abdom Radiol (NY).

[5] Miklos JR, Moore RD, Chinthakanan O (2015). Laparoscopic and robotic-assisted vesicovaginal fistula repair: a systematic review of the literature. J Minim Invasive Gynecol.

[6] Chinthakanan O, Sirisreetreerux P, Saraluck A (2023). Vesicovaginal fistulas: prevalence, impact, and management challenges. Medicina (Kaunas).

[7] Raji MO, Raji IA, Hassan M, Raji HO, Bashir AM, Suleiman IN, Abubakar HU (2021). Assessment of health-related quality of life of vesicovaginal fistula patients attending a repair center in Northwest Nigeria. Ann Afr Med.

[8] Moses RA, Ann Gormley E (2017). State of the art for treatment of vesicovaginal fistula. Curr Urol Rep.

[9] Mellano EM, Tarnay CM (2014). Management of genitourinary fistula. Curr Opin Obstet Gynecol.

[10] Creanga AA, Genadry RR (2007). Obstetric fistulas: a clinical review. Int J Gynaecol Obstet.

[11] Ríos‑Melgarejo C, García‑Nava RH, Castañón‑Hernández I, González‑Villegas HO (2020). [Nuestra experiencia tras la reparación laparoscópica de la fístula vesicovaginal]. Rev Mex Urol.

[12] Bazi T (2007). Spontaneous closure of vesicovaginal fistulas after bladder drainage alone: review of the evidence. Int Urogynecol J Pelvic Floor Dysfunct.

[13] Thompson JC, Halder GE, Jeppson PC (2024). Repair of vesicovaginal fistulae: a systematic review. Obstet Gynecol.

[14] Nezhat CH, Nezhat F, Nezhat C, Rottenberg H (1994). Laparoscopic repair of a vesicovaginal fistula: a case report. Obstet Gynecol.

[15] Sotelo R, Mariano MB, García-Segui A (2005). Laparoscopic repair of vesicovaginal fistula. J Urol.

[16] Porpiglia F, Fiori C, Morra I, Ragni F, Vaccino D, Scarpa RM (2009). Laparoscopic vesico-vaginal fistula repair: our experience and review of the literature. Surg Laparosc Endosc Percutan Tech.

[17] Zapata‑González JA, Dávila‑Cepeda A, García‑Sánchez SM, Reyna‑Bulnes A, Robles‑Scott M (2014). [Reparación laparoscópica de fístula vesicovaginal: nuestra experiencia]. Rev Mex Urol.

[18] Ockrim JL, Greenwell TJ, Foley CL, Wood DN, Shah PJ (2009). A tertiary experience of vesico-vaginal and urethro-vaginal fistula repair: factors predicting success. BJU Int.

